# Caudal DMN neurons innervate the spleen and release CART peptide to regulate neuroimmune function

**DOI:** 10.1186/s12974-023-02838-2

**Published:** 2023-07-04

**Authors:** Nobuhide Kobori, Anthony N. Moore, John B. Redell, Pramod K. Dash

**Affiliations:** Department of Neurobiology and Anatomy, The University of Texas McGovern Medical School, P.O. Box 20708, Houston, TX 77225 USA

**Keywords:** Brain–immune interaction, CART, Caudal DMN, Tract tracing

## Abstract

**Background:**

Inflammation is a fundamental biological response to injury and infection, which if unregulated can contribute to the pathophysiology of many diseases. The vagus nerve, which primarily originates from the dorsal motor nucleus (DMN), plays an important role in rapidly dampening inflammation by regulating splenic function. However, direct vagal innervation of the spleen, which houses the majority of immune and inflammatory cells, has not been established. As an alternative to direct innervation, an anti-inflammatory reflex pathway has been proposed which involves the vagus nerve, the sympathetic celiac ganglion, and the neurotransmitter norepinephrine. Although sympathetic regulation of inflammation has been shown, the interaction of the vagus nerve and the celiac ganglia requires a unique interaction of parasympathetic and sympathetic inputs, making this putative mechanism of brain–spleen interaction controversial.

**Body:**

As neuropeptides can be expressed at relatively high levels in neurons, we reasoned that DMN neuropeptide immunoreactivity could be used to determine their target innervation. Employing immunohistochemistry, subdiaphragmatic vagotomy, viral tract tracing, CRISPR-mediated knock-down, and functional assays, we show that cocaine and amphetamine-regulated transcript (CART) peptide-expressing projection neurons in the caudal DMN directly innervate the spleen. In response to lipopolysaccharide (LPS) stimulation, CART acts to reduce inflammation, an effect that can be augmented by intrasplenic administration of a synthetic CART peptide. These in vivo effects could be recapitulated in cultured splenocytes, suggesting that these cells express the as yet unidentified CART receptor(s).

**Conclusion:**

Our results provide evidence for direct connections between the caudal DMN and spleen. In addition to acetylcholine, these neurons express the neuropeptide CART that, once released, acts to suppress inflammation by acting directly upon splenocytes.

**Supplementary Information:**

The online version contains supplementary material available at 10.1186/s12974-023-02838-2.

## Introduction

Activation of the innate immune system and inflammatory responses are fundamental to how an organism responds to infection and injury [1]. Inflammation serves to limit the spread of infectious agents and to facilitate the removal of cellular debris in order to allow healing processes to proceed. Acute inflammation typically resolves once tissue homeostasis is restored. However, unresolved or exaggerated inflammation can hinder wound healing and negatively impact the pathobiology of many human diseases such as sepsis, rheumatoid arthritis, and neurodegenerative disease [2, 3]. Two mechanisms have been identified through which the brain can regulate inflammation to minimize the harmful consequences of exaggerated or protracted inflammation.

The classical pathway for regulating inflammation involves activation of the hypothalamic–pituitary–adrenal (HPA) axis. In this pathway, pro-inflammatory molecules released into the blood are detected by vagus afferents, and the information is transsynaptically transmitted to the hypothalamus. Stimulating the hypothalamus triggers the release of a chain of hormones, ending with the adrenal glands releasing cortisol into the blood stream. Cortisol attenuates peripheral inflammation by acting on inflammatory cells to reduce the synthesis and release of pro-inflammatory molecules [4]. In addition to this classical pathway, experimental studies aimed at controlling lipopolysaccharide (LPS)-induced inflammation (a widely used model to study the pathophysiology of inflammation) have shown that activation of the efferent component of the vagus nerve can rapidly reduce peripheral inflammation via regulation of splenocytes [5]. Though a role for the spleen in regulating disease- and trauma-associated inflammation has been established [6–8], the anatomical and electrophysiological evidence of connections between the vagus nerve and the spleen is controversial [9–11]. As a result, it has been proposed that vagus efferents make synaptic contact with the sympathetic celiac (alt. coeliac) ganglion. To complete the pathway, the splenic nerve originating from the celiac ganglia then innervates the spleen, where it releases norepinephrine onto splenocytes to reduce pro-inflammatory cytokine expression and release [12, 13]. While sympathetic regulation of inflammation has been repeatedly demonstrated, the proposed interaction of the vagus nerve and the celiac ganglia has not been widely accepted [14].

In the present study, we reasoned that neurons of the caudal DMN, which innervates lower body organs, may express neuropeptides that would enable us to follow their efferent axons to the lower organs, including the spleen. We show evidence demonstrating that cholinergic neurons within the caudal DMN express high levels of CART peptides (CARTp). Using a combination of immunohistochemistry, anterograde viral tract tracing, CRISPR-mediated knock-down, and functional assays, we also demonstrate that CART-expressing caudal DMN neurons directly innervate the spleen where CARTp is released and acts on splenocytes to dampen inflammation.

## Results

### CART peptide is expressed in the caudal DMN

**Fig. 1:**
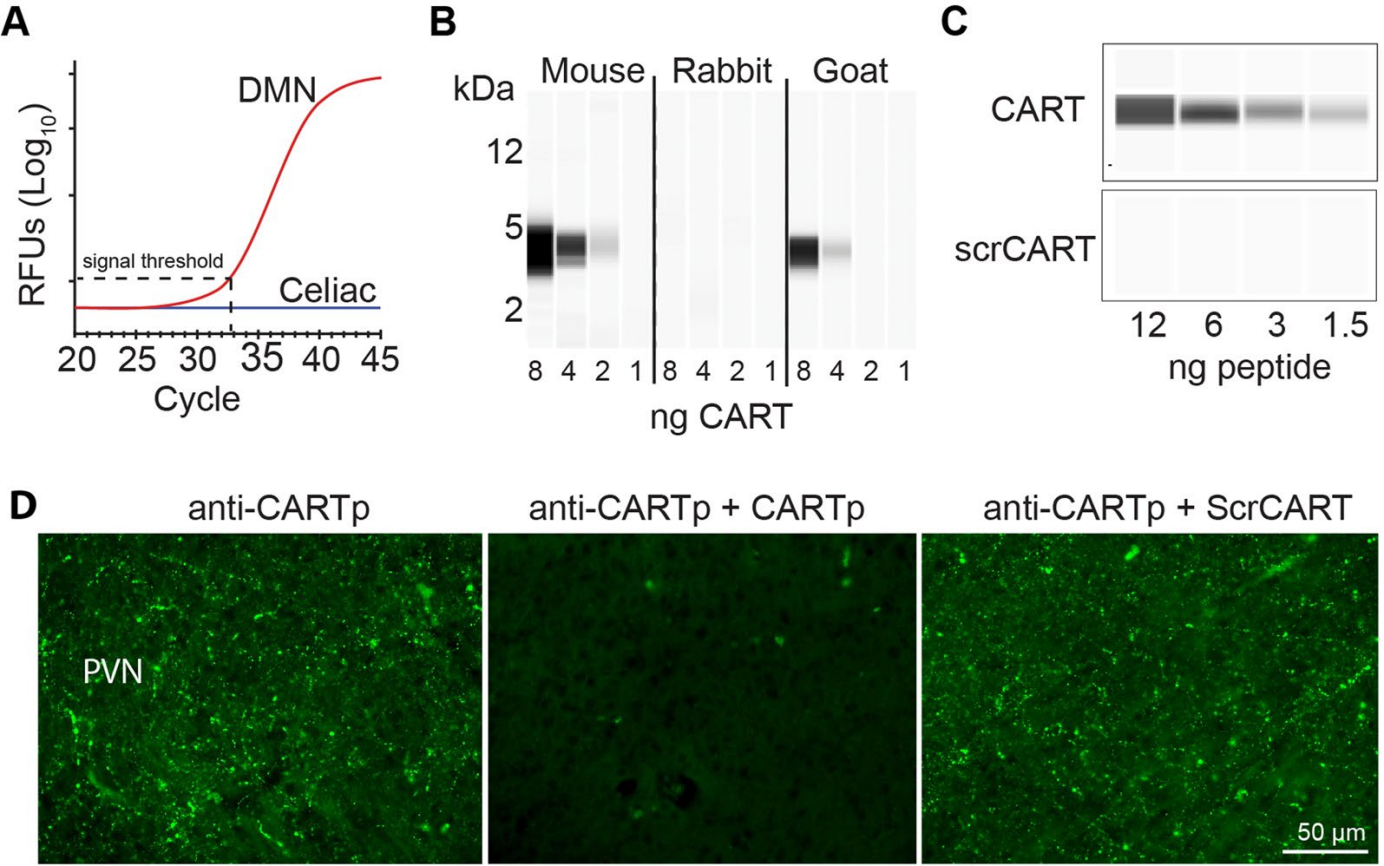
Characterization of CART peptide (CARTp) antibodies. **A** PCR amplification curves of CART mRNA extracted from donor cadaver DMN and celiac ganglion tissues. CARTp mRNA was detected in the DMN, but not in the celiac ganglion. **B** Representative capillary westerns showing CARTp immunoreactivity for three commercially available CART antibodies. Synthetic CARTp was denatured and loaded as the sample. **C** Representative capillary westerns showing the reactivity of the mouse anti-CARTp antibody against different amounts of synthetic CARTp and scrambled CARTp. **D** Representative images of the paraventricular nucleus (PVN, a site known to contain CART-positive axons) showing CARTp immunoreactivity in tissue sections incubated with either the mouse anti-CART antibody (CART Ab), the mouse anti-CART antibody after overnight neutralization with 30 molar excess CART peptide (CART Ab + CARTp), or the mouse anti-CART antibody after overnight neutralization with 30 molar excess scrambled CART peptide (CART Ab + Scr pep)

As neuropeptides are often expressed at high levels in neurons, we reasoned that neuropeptide immunoreactivity detected in DMN neurons could be used to examine vagus efferent innervation of the spleen. In order to identify candidate neuropeptides, we used micro-dissected human DMN tissues collected from donated cadavers to isolate total RNA and assess neuropeptide mRNA expression. Isolated celiac ganglia total RNA was used for comparison as this ganglia is the origin of the splenic nerve, which is known to project to the spleen. Figure 1A shows that cocaine- and amphetamine-regulated transcript preprotein (*CARTPT*) mRNA is expressed in the DMN, but was not detected in the celiac ganglia, suggesting that CARTp immunoreactivity may be useful to examine vagal projections to the spleen. Prior to examining CARTp expression using immunohistochemistry, we assessed the specificity of commercially available CARTp antibodies. Figure 1B shows representative immunoreactivity detected by capillary westerns using three commercially available CART antibodies and synthetic CARTp. Both the mouse (MAB163; R&D Systems) and goat (AF163; R&D Systems) antibodies recognized synthetic CARTp, while the rabbit antibody produced no specific signal. Further analysis of the mouse anti-CARTp antibody revealed that while it detected the synthetic CARTp, it did not cross-react with a control peptide containing a randomized CARTp amino acid sequence (referred to as scrambled CARTp, or scrCARTp; Fig. 1C). Figure 1D shows representative images of the paraventricular nucleus (PVN; a site known to contain CART-positive axons) demonstrating the functionality of the mouse anti-CARTp antibody for IHC. Specific immunoreactivity was attenuated when the primary antibody was pre-incubated with 30-fold molar excess CART peptide (anti-CARTp + CARTp), but not when pre-incubated with 30-fold molar excess scrambled peptide (anti-CARTp + scrCART). Based on these results, the mouse anti-CART antibody was used for all subsequent experiments.

**Fig. 2:**
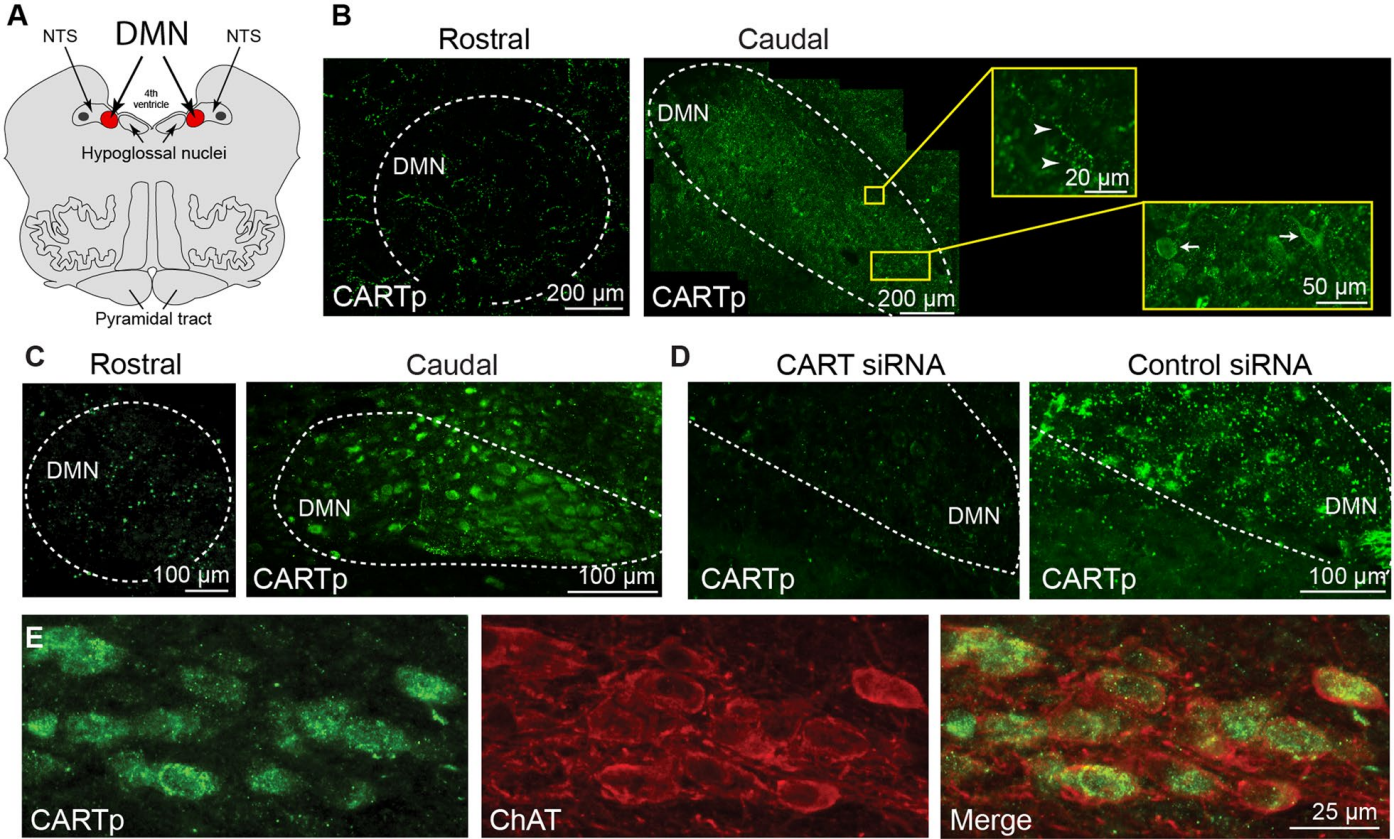
CART peptide (CARTp) is expressed in neurons in the caudal DMN. **A** Drawing of a coronal section of the human brain stem demonstrating the relative position of the DMN (rostral aspect in red) compared to the NTS and the 4th ventricle. **B** Representative images showing that CARTp immunoreactivity can be detected in neurons in the caudal, but not rostral, aspect of the DMN in humans. The insets show that both CARTp-positive cell somas (arrows) and fibers (arrowheads) can be seen in caudal DMN. **C** Representative mouse brain images showing CARTp immunoreactivity in neurons resident to the caudal, but not rostral, aspect of the DMN. **D** Intracerebroventricular administration of CART siRNA to mice significantly reduced CARTp immunoreactivity in the caudal DMN compared to control (non-targeting) siRNA. **E** Representative double-label immunohistochemistry showing co-localization of CARTp and ChAT immunoreactivities in the caudal DMN neurons in mice. Double immunostained cells appear yellow in the merged image

Brain stem tissues containing the DMN obtained from donated cadavers were used for evaluation of CARTp expression. The relative location of the DMN (Fig. 2A) was determined based on its position on the wall of the 4th ventricle (for the rostral DMN) and the location of the central canal (for the caudal DMN; not shown). When tissue sections containing the rostral DMN were stained for CARTp, only immunopositive fibers, but not cell bodies, were detected. In contrast, CARTp immunoreactivity was observed in both the soma (arrows) and fibers (arrowheads) of caudal DMN (Fig. 2B). This pattern of immunoreactivity appeared to be conserved across species as CARTp-positive neurons were also detected in the caudal, but not rostral, DMN of mice (Fig. 2C). To further corroborate the specificity of the CARTp immunostaining, 0.375 nmol of CART-specific or non-targeting siRNAs were injected into the lateral ventricle of mice (*n* = 3/group). This dose of siRNA was based on previous studies which administered siRNAs via intracerebroventricular infusion [15]. Forty-eight hours later, mice were euthanized and brain stem sections prepared and immunostained using the CARTp antibodies. Figure 2D shows representative images demonstrating that CARTp immunoreactivity was markedly reduced in mice that received CART-specific siRNA injection compared to the control siRNA. This was observed in all mice. Finally, as acetylcholine is the classical vagal neurotransmitter, we examined if choline acetyltransferase (ChAT), the enzyme responsible for acetylcholine biosynthesis, and CARTp immunoreactivities co-localized in the DMN cell bodies. Figure 2E shows representative images of double-stained mouse tissue sections containing the caudal DMN showing co-localization of CARTp (green) and ChAT (red) immunoreactivity in many cell bodies.

### CARTp-positive fibers innervate the spleen

**Fig. 3:**
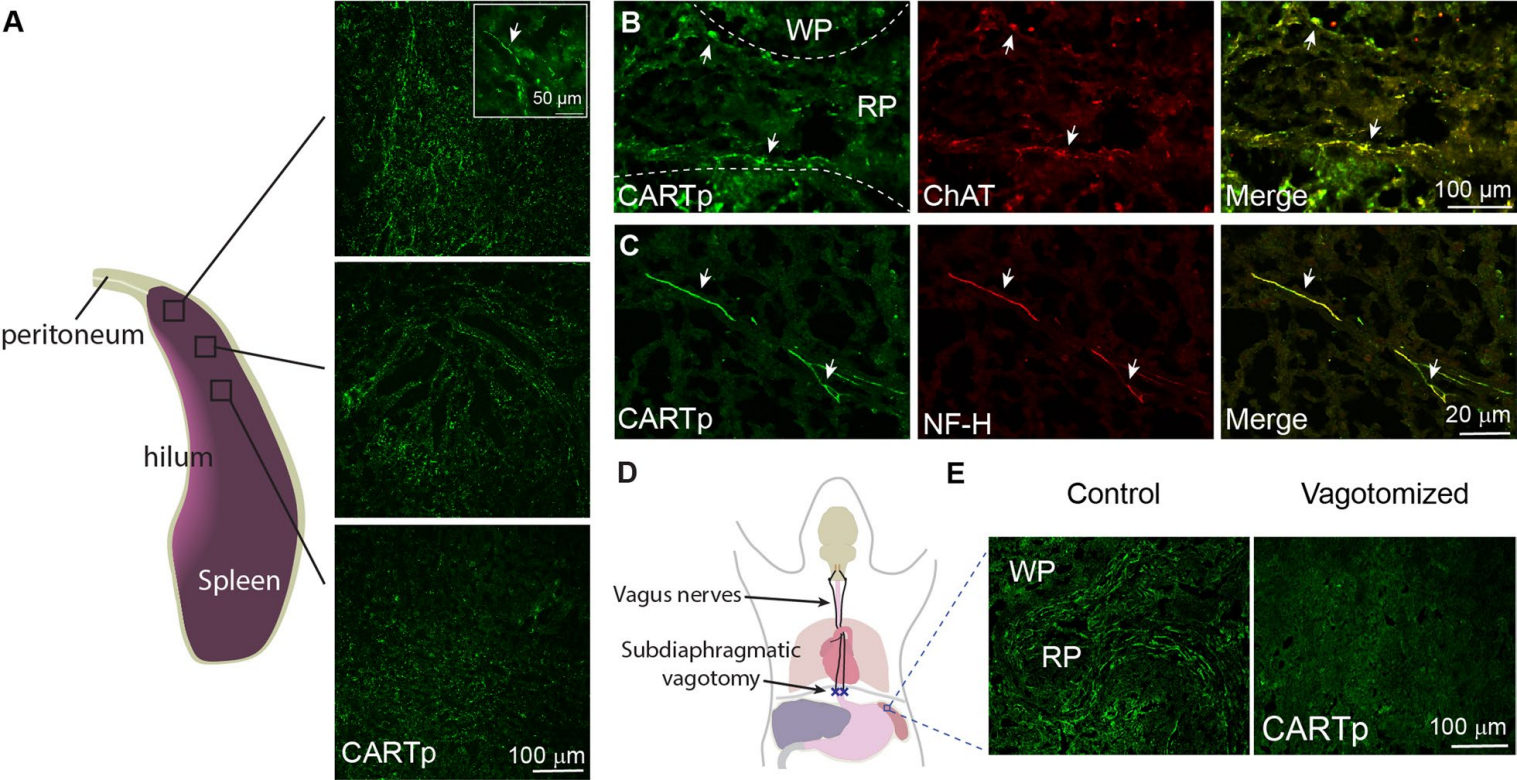
CARTp immunoreactivity is detected in the mouse spleen. **A** Representative confocal images showing CARTp immunoreactivity in the spleen. CARTp immunoreactivity appears as both fibers (inset, arrows) and puncta and could be found along the longitudinal axis of the spleen. **B** Representative photomicrographs showing that CARTp and ChAT immunoreactivities colocalize in fibers that appear primarily along the boundary of the red pulp (RP) adjacent to the white pulp (WP). **C** CARTp-immunoreactive fibers (arrows) co-localized with the axonal marker neurofilament H (NF-H). **D** Drawing illustrating the relative position of the subdiaphragmatic vagotomy. **E** Representative images showing that four weeks following subdiaphragmatic vagotomy CARTp immunoreactivity in the spleen is lost

As neurons in the caudal DMN send efferent projections to innervate lower peripheral organs, our observation that CARTp is expressed in DMN neurons suggests that CARTp immunoreactivity may be a useful marker of vagal innervation of the spleen. Representative confocal images of CARTp-immunostained spleen sections are shown in Fig. 3A. CARTp-positive fibers could be observed along the boundaries of the red pulp and were most concentrated at the apical tip, becoming less dense along the longitudinal axis of the spleen. CARTp immunoreactivity in the spleen co-localized with ChAT (Fig. 3B), as was observed in the caudal DMN, as well as with the axonal marker neurofilament-H (NF-H, Fig. 3C). To determine if severing the vagus nerve eliminated CARTp immunoreactivity in the spleen, rats with bilateral subdiaphragmatic vagotomy (Fig. 3D) or control sham surgery were purchased from Charles River Laboratories Surgical Services (*n* = 4/group). Rats were used for this study as we have observed CARTp-expressing neurons in the rat caudal DMN, and mice with bilateral subdiaphragmatic vagotomy were not commercially available. To allow time for Wallerian degeneration of the distal vagus nerve, spleens were removed and CARTp immunoreactivity examined 4 weeks after the surgery. Representative images of CARTp-immunostained spleen sections from control and vagotomized animals are shown in Fig. 3E. Compared to sections from control rats, vagotomy resulted in a loss of CARTp-immunoreactive signal in the spleen. This was observed in all animals, indicating that the CARTp-positive fibers observed in the spleen could be direct projections of the vagus nerve. This innervation does not appear to enter the spleen via the splenic nerve, as no CART-positive fibers were observed in conjunction with tyrosine hydroxylase (TH)-positive sympathetic fibers (Additional file 1: Fig. S1), consistent with our observation that CART mRNA was not present in the celiac ganglia.

### Anterograde viruses injected into the caudal DMN, but not the rostral DMN, reveals direct innervation of the spleen

**Fig. 4:**
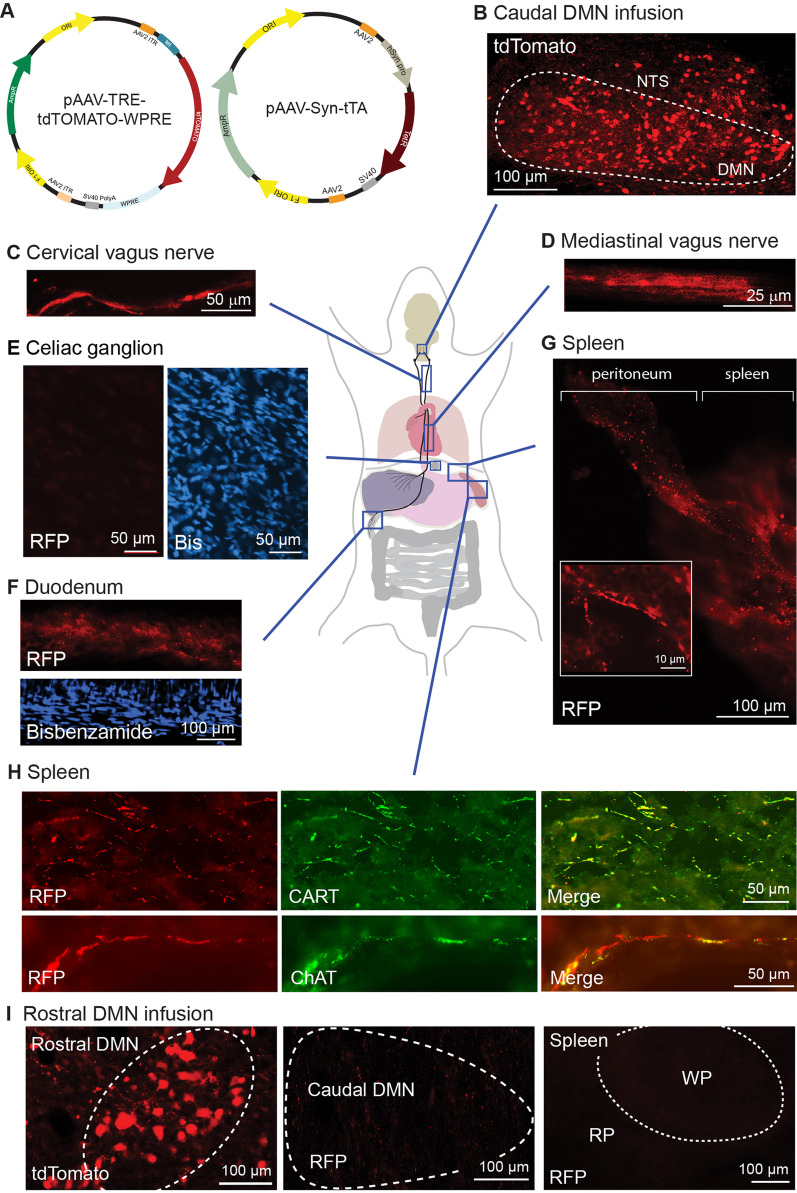
Anterograde labeling of caudal DMN efferents can be tracked directly to the spleen. **A** Schematic drawing of the plasmids separately packaged into AAV9 and used to direct neuronal expression of tdTomato for tract tracing. **B** Representative photomicrographs showing tdTomato expression 30 days after virus injection in the mouse caudal DMN. The continuity of tdTomato signal could be observed in the vagus efferents present at the **C** cervical and **D** mediastinal (between the lungs) regions. **E** No tdTomato signal (or RFP immunoreactivity) was detected in the celiac ganglia, the source of the splenic nerve. **F** tdTomato immunopositive (detected using anti-RFP) vagal efferents were seen in the duodenum (a site of known vagal innervation). **G** Montage image showing that tdTomato could be tracked from the peritoneum near the upper medial tip of the spleen, emerging into the parenchyma. Inset, High magnification (100 µm width). **H** Representative images showing nerves containing tdTomato- and CARTp-positive (and tdTomato- and ChAT-positive) fibers along the boundary of the white pulp of spleen. **I** When viral injection was targeted to the rostral DMN, tdTomato could be detected in the rostral DMN, but not the caudal DMN or the spleen

Although our vagotomy experiments were performed in rats, it is anticipated that similar results would have been observed in mice. To confirm that the spleen is directly innervated by DMN neurons in the mouse, we co-injected a mixture of two viral constructs (AAV9-TRE-tdTomato and AAV9-Syn-tTA, Fig. 4A) bilaterally into the caudal DMN of mice (*n* = 6). Expression of the tetracycline transactivator protein (tTA) is driven by the neuron-specific synapsin 1 promoter and acts in trans to induce expression of tdTomato only in DMN neurons transduced by both viruses [16]. Figure 4B shows the expression of tdTomato surrounding the injection site in the caudal DMN. tdTomato expression was largely restricted to the caudal DMN, with expression observed in the adjacent nucleus tractus solitarius (NTS) in some animals. In order to verify that the injected viral constructs did not cross synapses as has been observed with high titers of AAV1 (and to a lesser extent AAV9), we examined tdTomato expression in the medial parabrachial nucleus (PBN) and paraventricular nucleus (PVN) (Additional file 1: Fig. S2), structures that receive monosynaptic input from the DMN and/or NTS [17–19]. Consistent with previous studies indicating that most AAV isotypes do not cross synapses, we observed tdTomato in presynaptic fibers within the PBN and PVN, but not in PBN/PVN cell bodies [20].

To trace tdTomato from the caudal DMN to innervated peripheral organs, we used an anti-red fluorescence protein (RFP) antibody (tdTomato is a form of RFP) to amplify the signal. We observed anti-RFP immunoreactivity in the vagus nerve at the level of the cervical spine (Fig. 4C) and just above the diaphragm in the lower mediastinal region (Fig. 4D). RFP immunoreactivity was not observed in the celiac ganglia (Fig. 4E), but was detected in the duodenum of the small intestine, a site known to receive direct vagal input (Fig. 4F) [21]. RFP immunoreactivity was detected inside the peritoneum as it attaches to the apex of the spleen, as well as in the spleen parenchyma (Fig. 4G). To assess if the RFP immunoreactivity in the spleen co-localized with CARTp (and in independent sections, ChAT), double-label immunohistochemistry was performed. Figure 4H shows that fibers immunopositive for both tdTomato and either CARTp or ChAT could be observed in the spleen. These projections did not originate from structures known to innervate the spleen including the dorsal root ganglia or nodose ganglia, as no RFP immunostaining was observed in these ganglia (Additional file 1: Fig. S2). When the virus constructs were injected into the rostral DMN (Fig. 4I), no tdTomato signal (or RFP immunoreactivity) was observed in either the caudal DMN or the spleen. Our observations of tdTomato distribution (either the fluorophore signal or anti-RFP immunoreactivity) following DMN injection were consistent across all animals examined (*n* = 6). Taken together, these findings demonstrate that CARTp-expressing neurons in the caudal DMN send direct axonal projections that innervate the spleen.

### CARTp regulates inflammation

**Fig. 5:**
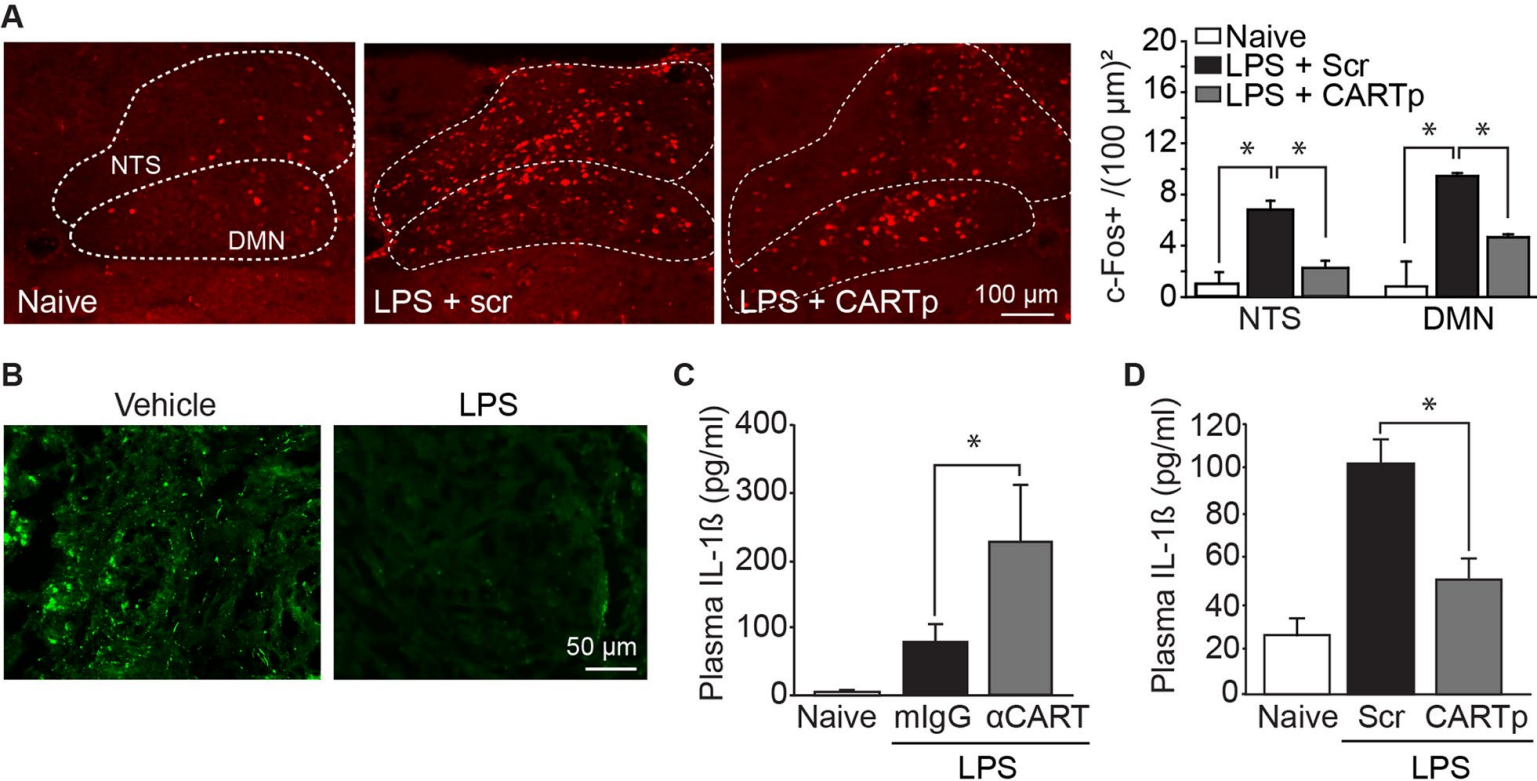
CARTp dampens LPS-triggered inflammation. **A** Representative photomicrographs showing c-Fos immunoreactivity, and summary c-Fos positive cell counts, in the caudal DMN and NTS of mice treated with either scrambled CARTp or CARTp followed by LPS administration. Uninjected controls are shown for comparison. **B** Representative images showing CARTp immunoreactivity in the spleen in vehicle- and LPS-treated mice. LPS causes a decrease in CARTp immunoreactivity, presumably due to its release from vagal efferents. **C** Summary data showing the plasma levels of IL-1ß in mice 3 h after injection with LPS given following intrasplenic infusion of either 9 µg/kg control IgG or 9 µg/kg anti-CART antibodies. Uninjected mice were used as baseline controls. **D** Summary data showing the levels of IL-1ß in the plasma of mice 3 h after injection with LPS following intrasplenic infusion of either 3.8 µg/kg scrambled CARTp or 3.8 µg/kg synthetic CARTp. Uninjected mice were used as baseline controls. Data are presented as the mean ± SEM. *, *p* < 0.05

The LPS injection model has been widely used to study the innate immune response, neuroinflammation, and the role of the vagus system in the regulation of inflammation [22–24]. Previous studies have shown that increased plasma levels of pro-inflammatory cytokines in response to LPS (and other agents) results in increased neuronal activity within the NTS and DMN, which can be detected by examining the activity marker c-Fos [25]. To examine if CARTp (like acetylcholine) has anti-inflammatory effects, we first examined c-Fos expression in the NTS and DMN in response to treatment with LPS ± CARTp. A dose of 650 µg/kg CARTp was used based on previous studies examining the effect of CARTp on various behaviors [26, 27]. The summary data (*F* = 29.192, *p* < 0.001; *n* = 4/group) presented in Fig. 5A show that c-Fos expression in the NTS and DMN was significantly increased in mice treated with 85 µg/kg LPS + 650 µg/kg control peptide (scrambled CARTp). This increase was significantly reduced in mice that received 85 µg/kg LPS + 650 µg/kg CARTp, suggesting that CARTp treatment may reduce inflammation. As our evidence shows that CARTp is expressed in the cell bodies of the DMN and then transported to axon terminals in the spleen, we anticipated that LPS treatment would cause release of CARTp in the spleen, resulting in a reduction in immunoreactivity. As expected, Fig. 5B shows that CARTp immunoreactivity in the spleen is decreased 3 h after LPS exposure.

To directly examine if CARTp released into the spleen in response to LPS can limit the inflammatory response, a mouse monoclonal CARTp neutralizing antibody (9 µg/kg), or an equal amount of a normal mouse IgG, was injected directly into the spleen 15 min prior to LPS challenge (*n* = 4/group). Three hours later, plasma was collected for measuring the levels of the pro-inflammatory cytokine IL-1β in circulation. The summary data presented in Fig. 5C show that administering CARTp neutralizing antibodies directly into the spleen significantly exacerbated the IL-1ß response to LPS (*F* = 8.028, *p* = 0.005). In contrast, a significant decrease in plasma IL-1ß was observed when 0.4 ng exogenous CARTp, but not a control scrambled CARTp peptide (Scr), was infused directly into the spleens of separate groups of mice (*n* = 4/group; *F* = 8.598, *p* = 0.004; Fig. 5D). This dose was based on the systemic dose after correction for the mass of the spleen relative to total body weight. When 0.4 ng CARTp was administered i.v., no significant reduction in plasma IL-1ß levels were observed (data not shown), indicating that peptide entering the circulation from the splenic injections cannot explain the reduced plasma IL-1ß levels we observed.

### Deleting *Cartpt* gene in caudal DMN neurons reduces CARTp in the spleen and exacerbates the inflammatory response to LPS

**Fig. 6:**
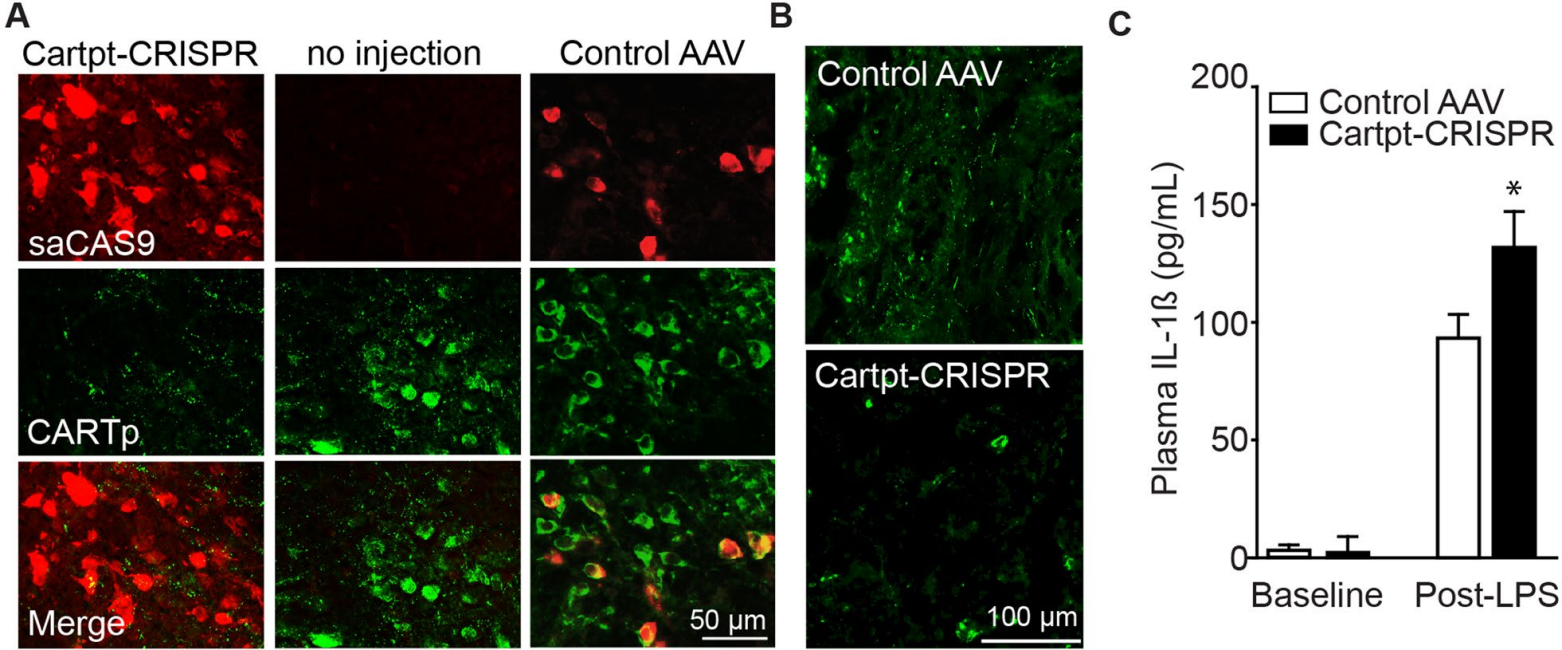
CRISPR-Cas deletion of the Cartpt gene from the DMN exacerbates LPS-induced systemic inflammation. **A** Representative photomicrographs of saCAS9 (a RNA-guided DNA endonuclease) and CARTp immunoreactivities in the caudal DMN of mice injected with either *Cartpt*-CRISPR or a control AAV. *Cartpt*-CRISPR effectively reduced CART expression in the caudal DMN and **B** the spleen as compared to mice receiving the control AAV. **C** Summary data showing the circulating levels of IL-1ß in mice receiving *Cartpt*-CRISPR or control AAV infusions into the caudal DMN followed by injection of LPS (or vehicle). Loss of CART exacerbated circulating IL-1ß levels

While the above findings show that CARTp in the spleen participates in reducing plasma IL-1ß levels after LPS stimulation, they do not directly address whether or not CARTp expressed in DMN neurons is necessary. We therefore deleted the *Cartpt* gene in caudal DMN neurons using a viral CRISPR-saCas9 AAV construct. The *Cartpt*-CRISPR virus, or a control AAV virus lacking the *Cartpt* guide sequence, was injected into the right caudal DMN of mice (*n* = 3), while the left caudal DMN was uninjected. Two months later, animals were perfusion-fixed and tissue sections containing the caudal DMN were double-immunostained using anti-Staphylococcus aureus CAS9 (saCAS9) and anti-CARTp antibodies. Figure 6A shows robust saCAS9 immunoreactivity in the DMN of mice that received the *Cartpt*-CRISPR-AAV, concomitant with reduced CARTp immunoreactivity. In contrast, the uninjected contralateral DMN from the same animals showed numerous CARTp-positive cells. In separate mice injected with the control AAV construct (*n* = 3), transduced cells immunopositive for both saCAS9 and CARTp could be observed. The spleens of mice that received bilateral caudal DMN injections of the *Cartpt*-CRISPR AAV showed reduced CARTp-immunoreactive fibers (Fig. 6C) as compared to mice that received bilateral injections of the control AAV. A few CARTp-positive fibers could still be observed in the *Cartpt*-CRISPR AAV treated mice, possibly due to incomplete transduction of both caudal DMNs. In separate groups of animals (*n* = 3/group), mice that received bilateral caudal intra-DMN *Cartpt*-CRISPR-AAV had significantly higher LPS-induced plasma levels of IL-1ß as compared to mice that received control virus (interaction of LPS and treatment: *F* = 12.407, *p* = 0.004) (Fig. 6C).

### CARTp acts directly on immune cells

**Fig. 7:**
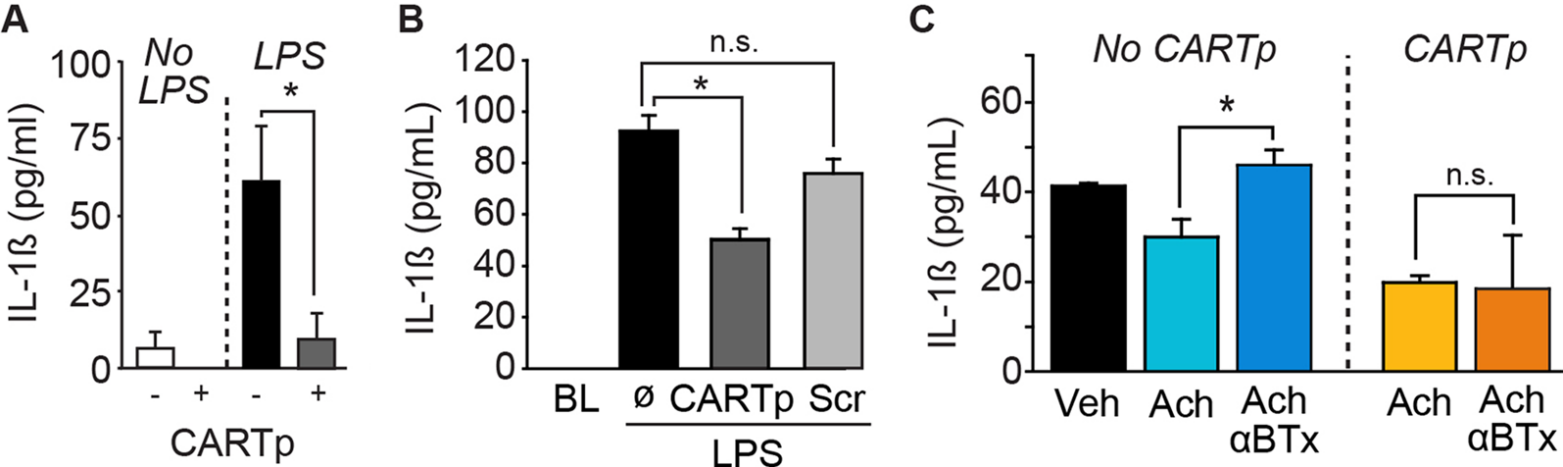
CARTp can reduce inflammation independent of nicotinic cholinergic receptors. **A** Summary data showing that IL-1ß levels are significantly enhanced in splenocyte culture media in response to LPS. CARTp treatment significantly reduced IL-1ß levels. **B** Summary data showing that the LPS-mediated increase in IL-1ß in RAW264.7 cell media (*n* = 5/group) is significantly reduced by treatment with CARTp, but not by the control scrambled CART peptide. Ø, no treatment. **C** Summary results showing that treatment with the nicotinic acetylcholine receptor blocker α-bungarotoxin blocked the anti-inflammatory effect of acetylcholine, but was ineffective at blocking the effect of CARTp treatment. This suggests that CARTp can reduced inflammation independent of nicotinic cholinergic receptors

To address the possibility that immune cells within the spleen can directly respond to CARTp, splenocytes were isolated and then treated with either 8 µg/ml CARTp or an equal amount of a scrambled CART sequence peptide. Fifteen minutes later, splenocytes were exposed to LPS (5 µg/ml) or vehicle (*n* = 5/group). Figure 7A shows that the IL-1ß level in the culture media was significantly reduced in response to CART peptide as compared to media taken from control splenocytes (*t* = − 2.954, *p* = 0.0418). CARTp treatment also reduced IL-1ß levels in non-LPS treated cells, although this reduction did not reach statistical significance (*p* = 0.186). Similar to that seen in splenocytes, mouse macrophages (RAW264.7 cells; *n* = 5/group) responded to LPS and released IL-1ß (*F* = 24.982, *p* < 0.001), an effect that was significantly reduced by pretreatment with CARTp (t = 7.007, *p* < 0.001), but not by the scrambled CART peptide (Fig. 7B). As both CARTp and acetylcholine are present in DMN nerve terminals where they can be released in response to LPS, we questioned if the anti-inflammatory action of CARTp is dependent on nicotinic acetylcholine receptor activation. Figure 7C shows that while the reduction in IL-1ß levels in response to acetylcholine (Ach) could be blocked by the nicotinic receptor blocker α-bungarotoxin (αBTx; *F* = 5.35, *p* = 0.022; Ach vs Ach + αBTx: *t* = 3.241, *p* = 0.021), the CARTp-mediated reduction in IL-1ß was unaffected by αBTx (*H* = 6.031, *p* = 0.040; CARTp vs CARTp + αBTx: *Q* = 0.766, *p* = 1.000). This suggests that CARTp is capable of suppressing inflammation even when nicotinic receptors are inhibited.

## Discussion

It has been previously demonstrated that vagus nerve stimulation exerts an anti-inflammatory response. It has been postulated that the vagus nerve regulates inflammation via engagement of the sympathetic celiac ganglion and splenic nerve, although direct evidence for this is lacking. In the present study, we identified CARTp as a neuropeptide which is expressed in the caudal DMN, and using CARTp immunoreactivity, vagotomy, tract tracing, and CRISPR-Cas knock-down, we provided evidence that the caudal DMN directly innervates the spleen.

Previous studies to identify direct vagal innervation of the spleen have yielded contradictory results. For example, Bellinger et al., found no evidence of cholinergic innervation in the mouse spleen using imaging based on the conversion of acetyl-[^14^C]coenzyme A to Ach [28]. Using ChATBAC-eGFP mice that express GFP in cholinergic neurons, Kaestner et al., reported the presence of cholinergic terminals around the cell bodies of the celiac ganglion, but limited staining in the spleen. This cholinergic input does not appear to be vagal in nature, as Bratton et al., have recently demonstrated that vagal stimulation does not influence splenic nerve or suprarenal ganglion activity [10]. In contrast to these findings, Buijs et al., using pseudorabies virus, and Cailotto et al., using cholera toxin B, have demonstrated that splenic injection of these agents leads to neuronal labeling within the DMN [29, 30], effects that can be blocked by denervation of the splenic apices. Consistent with this, Guyot et al., demonstrated the presence of apical splenic innervation in mice that, when stimulated, released Ach and reduced inflammation [31].

Our results show that the spleen is directly innervated by CARTp-positive efferent axons originating from the caudal DMN, and that these fibers travel to the apical spleen from a branch of the vagus within the peritoneum. To the best of our knowledge, this is the first study to utilize anterograde viral tracers targeted to the DMN to examine if vagal efferents project to the spleen. In response to LPS injection, these vagus efferents release CART, (likely in conjunction with acetylcholine) where it acts on splenocytes to dampen their inflammatory response. Our findings demonstrate an endogenous pathway through which CART peptides can regulate peripheral inflammation, and provides a mechanistic explanation for a previous report that indicated that systemic administration of CART peptides can reduce pro-inflammatory cytokine expression after middle cerebral artery occlusion [32].

Our in vitro studies indicate that CARTp can act directly on macrophages (RAW264.7 cells) to reduce Il-1ß levels even in the presence of the nicotinic receptor blocker α-bungarotoxin, indicating that CARTp’s anti-inflammatory effect is independent of α7nAChR function. However, as vagus nerve activity is likely to release both CARTp and ACh, they may act in concert to suppress inflammation. A few caveats to our study remain to be addressed. First, all of our mice were male, and as the inflammatory response is influenced by sex [33–35], we are uncertain if the doses of synthetic CARTp used in this study would have similar effects in females. As a bona fide receptor (or receptors) for CARTp has yet to be identified, we are uncertain as to which cells are directly influenced by CARTp. In cultured splenocytes and RAW264.7 cells, we observed a significant decrease in IL-1ß production in response to CARTp, suggesting that monocytes/macrophages may be the site of action. While our intrasplenic infusion experiments demonstrate a role for CARTp in regulating inflammation mediated by the spleen, we cannot rule out that CARTp (both endogenous and systemically administered synthetic) may have influences on other inflammatory pathways, such as the intestinal cholinergic anti-inflammatory pathway [36]. Unlike Ach, which is rapidly degraded after its release, CARTp has been reported to have a serum half-life of about 6 h. A long serum half-life may enable released CARTp to suppress inflammatory response for an extended time period. This also suggests that a CART-based therapy may have a lasting benefit, reducing the necessity for chronic treatment paradigms that are more likely to be associated with unwanted side-effects such as weight loss.

## Conclusions

The present study demonstrates that CARTp is synthesized by vagus motor neurons and its expression can be used to identify vagal input to the spleen. CARTp plays a prominent role in decreasing inflammation, an effect mediated by release of CARTp into the spleen where it acts on splenocytes expressing a yet to be identified CARTp receptor. Exogenously administered CARTp or peripherally acting CARTp receptor agonist may have therapeutic values for treating inflammatory diseases as well as diseases such as traumatic brain injury, stroke, and Alzheimer’s disease where inflammation contributes to disease pathology.

## Methods

### Materials

CART peptide (CARTp) and control scrambled CARTp were synthesized and purified by GeneMed Synthesis (San Antonio, TX). The active CARTp sequence from amino acid 55–102 of the rat prepropeptide (accession #NP_058806) was allowed to fold naturally to form disulfide bridges between the cysteine residues. The scrambled CARTp has a reverse amino acid sequence from the C-terminal to N-terminal of the CART peptide. The biological activity of synthesized CARTp was confirmed by comparison to peptides purchased from Tocris Bioscience (Minneapolis, MN). Mouse anti-CART (MAB163) and goat anti-CART (AF163) antibodies were purchased from R&D Systems (Minneapolis, MN). Anti-tyrosine hydroxylase (AB152) antibodies were obtained from Sigma-Aldrich (St. Louis, MO). Anti-saCas9 (ab203933) and anti-choline acetyltransferase (ChAT, ab178850 and ab221793) antibodies were purchased from Abcam (Cambridge, UK). Anti-red fluorescent protein antibody (RFP, 600-401-379) was purchased from Rockland Immunochemicals (Pottstown, PA). Neurofilament-H (PA1-10002) antibody was purchased from ThermoFisher (Waltham, MA). Cadaver tissue was made available as a result of a whole body donation to the *Willed Body Program* of the UTHealth-McGovern Medical School.

### Animals and drug treatments

C57BL/6 male mice (12 weeks of age; single housed) and male SD rats (12 weeks of age; group housed) were maintained on a 12-h light/dark cycle, with ad libitum access to food and water. Experiments were performed during the light cycle. All experimental procedures were conducted in accordance with the Guide for the Care and Use of Laboratory Animals of the National Institutes of Health and approved by the Institutional Animal Care and Use Committee (IACUC). LPS was prepared by dissolving in PBS to a concentration of 1 mg/mL and then injected (i.p.) at a dose of 85 µg/kg. CARTp (or scrambled CARTp) was prepared in PBS to a concentration of 200 µg/mL and then injected (i.v.) at doses indicated in the figures.

### Rat vagotomy

Male Sprague Dawley rats that received bilateral subdiaphragmatic vagotomy or sham surgery (age-matched control rats) were purchased from Charles River Laboratories Surgical Services (Wilmington, MA). According to the vendor, rats were anesthetized with ketamine/xylazine then a midline abdominal incision was made and the liver and stomach retracted to expose the diaphragm. Each of the vagus nerve trunks were carefully isolated below the diaphragm and 2–3 mm section of each trunk excised. The abdominal incision was closed with sutures and the skin closed with wound clips. Rats were given buprenorphine and carprofen as analgesics. Once the rats arrived at UTHealth, they were housed for one month prior to use. Rats were then anesthetized with sodium pentobarbital (100 mg/kg), and transcardially perfused with cold saline followed by 4% paraformaldehyde. Brains and spleens were removed and used for immunohistochemistry.

### siRNA infusion

CART siRNA (Silencer Select® 4390771: s77585) and negative control siRNA (Silencer Select® 4390843) were purchased from Invitrogen. CARTPT siRNA and control siRNA were reconstituted in DEPC-treated water at a concentration of 1 mM and packaged into *Invivofectamine* (Invitrogen) using the methods recommended by the manufacturer. Mice were deeply anesthetized using sodium pentobarbital and mounted on the stereotaxic frame and 0.375 nmol of siRNA (0.5 µl) /mouse was injected into the right lateral ventricle.

### Immunohistochemistry and cell counts

At the indicated time points, rats or mice were deeply anesthetized using sodium pentobarbital. Once the animal failed to respond to tail and foot pinch, it was transcardially perfused with ice-cold PBS followed by 4% paraformaldehyde in PBS. The tissues were removed and post-fixed in 4% paraformaldehyde overnight, followed by incubation in 30% sucrose at 4 °C for 5 days before freezing in OCT solution (Fisher Scientific, Hampton, NH). 15-µm- to 30-µm-thick cryosections were collected in PBS (or directly mounted on glass slides, when necessary). The location of the DMN in brain sections was determined based on its anatomical position, and the result of cresyl violet staining. Sections were treated with 0.3% Triton X-100 and 2% normal goat serum in PBS and incubated in the primary antibody overnight in 4 °C. The fluorescent signal was detected with Alexa-488-, Alexa-568-, or Alexa-647-conjugated and highly cross-species-absorbed secondary antibodies (Invitrogen, Waltham, MA). Sections were treated with bisbenzamide (1 µg/ml) in order to visualize either the boundaries of the DMN and NTS (for brain stem sections), or the peritoneum, red pulp, and white pulp in spleen sections. Images were collected on either a Zeiss Axiovert microscope equipped with a Retiga 6000 camera or a Nikon A1R confocal microscope. Imaging settings were maintained across groups.

For cell counts, 30-µm-thick axial cryosections containing the caudal DMN (every 360 µm) were immunostained with an anti-cFos antibody (Sigma-Aldrich) and counterstained with bisbenzamide. Images were acquired using a Zeiss Axiovert microscope equipped with a Retiga 6000 camera. Camera settings were established using LPS-treated tissues, and then held constant across all images for that experiment. The DMN and NTS were carefully outlined using bisbenzamide staining to help identify brain stem nuclei, and the number of cFos-immunopositive cells counted by 2 independent investigators who were blind to the experimental groups.

### Blood collection and plasma preparation

At the indicated time points, mice were deeply anesthetized with 100 mg/kg sodium pentobarbital. Once the animal failed to respond to tail and foot pinch, the heart was exposed and blood was collected by cardiac puncture using a 21 Ga blunt-end needle attached to a 1-ml syringe. EDTA was added to the syringe as the anti-coagulant. Platelet-poor plasma was prepared by centrifuging the blood at 1000 × *g* for 10 min to remove the erythrocytes, leucocytes, and platelets. The supernatant solution was removed, and centrifuged again at 10,000 × *g* for 10 min to generate a platelet-poor plasma fraction. Plasma was aliquoted and frozen at − 80 °C until needed.

### Enzyme-linked immunosorbent assays (ELISAs)

Plasma IL-1ß levels were assessed using DuoSet ELISA kits (R&D systems) as instructed by the vendor. Plasma CARTp levels were assessed using a CART EIA kit (MilliporeSigma, Burlington, MA). The range of the standards was based on the vendors' instructions and on our previous experience with these techniques. The concentrations of target protein each sample (assayed in triplicate) were calculated by comparison to the appropriate reference standard curve.

### Intra-splenic infusions

C57BL/6 male mice were deeply anesthetized with isoflurane and then prepared for sterile survival surgery using alternating scrubs of ethanol and betadine. Prior to incision, the site was infused with 0.25% bupivacaine as an analgesic. A 0.5-cm incision was made on the left side and the spleen isolated as described previously [37]. A Hamilton syringe was used to inject the spleen at 3 sites along its axis with either anti-CART antibodies (9.0 µg/kg), control IgG (9.0 µg/kg), CARTp (3.8 µg/kg), or scrambled CARTp (3.8 µg/kg). Total injection volume was maintained at 60 µl. After completing the injections, the incision was sutured, and a topical antibiotic was applied.

### Splenocyte isolation and culture

Mice were deeply anesthetized with 100 mg/kg sodium pentobarbital. The spleen was removed and splenocytes dissociated using a Spleen Dissociation Kit (Miltenyi Biotec, Bergisch Gladbach, Germany) following the manufacturer’s protocol. The isolated splenocytes were cultured for 72 h in RPMI1640 medium (high glucose, Thermo Fisher) with 25 mM HEPES, 1 × GlutaMAX® (2 mM glutamine, Thermo Fisher), 1 × non-essential amino acids (NEAA), 1 mM sodium pyruvate, 1 × Anti-Anti ® (mixture of antibiotics and antimycotics, Thermo Fisher), and 10% heat-inactivated fetal bovine serum (FBS). After 72 h, the splenocytes were collected by centrifugation at 300 × *g* for 3 min at 10 °C then washed with RPMI1640 medium. The cells were counted and seeded onto 24-well plates at a density of 1 × 10^6^ cells/mL (1 mL/well) in RPMI1640 medium with 25 mM HEPES, 1 × GlutaMAX®, 1 × NEAA, 1 mM sodium pyruvate, 1 × Anti-Anti®, and 2% FBS, and incubated at 37 °C for 6 h. CARTp (8 µg/mL) or scrambled CARTp (8 µg/mL) were added to the culture medium, and then 15 min later, LPS (5 µg/mL, or vehicle) was added. The splenocytes were incubated for 9 h at 37 °C. The culture medium was centrifuged at 1000 × *g* for 10 min at 4 °C and the supernatant solutions collected and frozen until assayed.

### RAW264.7 cultures

RAW264.7 cells were maintained in Dulbecco’s modified Eagle medium (DMEM) supplemented with GlutaMax®, Anti-Anti® (antimycotics and antibiotics), and 10% FBS (Gibco/ThermoFisher) in 75-cm^2^ flask at 37 °C and 5% CO_2_. Every 5 – 7 days, the cells were re-plated by detaching the cells with 0.05% trypsin–EDTA for 5 min and gentle scraping, followed by centrifugation at 1500 × *g* for 8 min. For experimental use, the cells were plated in either 24-well culture plates at 5.0 × 10^5^ cells/well or in 96-well culture plates at 1.0 × 10^5^ cells/well. Twenty-four hours later, 50 µl culture medium/well was replaced by fresh medium which contained experimental treatments. The final concentrations of the experimental treatments were 5 µg/ml LPS, 8 µg/ml CARTp or scrambled CARTp, acetylcholine (Ach) 50 µM, and α-bungarotoxin (αBtx) 50 µM. The cells were incubated in the experimental medium for 24 h. The culture medium was collected and stored in − 20 °C until assayed for IL-1ß by ELISA.

### Viral injection and tract tracing

For producing the tdTomato expression viruses, pAAV-TRE-tdTomato-WPRE and pAAV-Syn1-tTA plasmids were obtained from Addgene (https://doi.org/10.7554/eLife.40350). The plasmids were amplified and packaged separately into AAV9 by the Gene Vector Core of Baylor College of Medicine (Houston, TX). These two viruses were mixed immediately prior to infusion. The final titer of the viruses was adjusted to 1 × 10^10^ infectious units/mL for tdTomato virus and 1 × 10^9^ infectious units/mL for Syn1-tTA virus. Injected mice were housed for 1 month post-surgery to allow time for the expressed tdTomato to fill the cell bodies and axons. The *Cartpt*-CRISPR RNA (crRNA) sequence was selected from several candidates within the initial segment of the CARTp-coding region of *Cartpt* using the software available at https://github.com/aryeelab/guideseq#start-of-content (https://doi.org/10.1038/nbt.3117). To generate the CRISPR-Cas9 vector, pX601_AAV plasmid, which contains the staphylococcus aureus Cas9 expression cassette under the control of the CMV promoter, was used. DNA which coded the guide RNA (gRNA) was inserted into the pX601_AAV plasmid under the control of the U6 promoter by GeneScript (Piscataway, NJ). The sequence of the *Cartpt* crRNA was 5’-GCGTGGGACGCATCATCCACGG-3’. A scrambled sequence was used to develop a control vector. These vectors were packaged into AAV9 by the Baylor College of Medicine gene vector core. The titers of the SynaptoTag AAV and *Cartpt*-CRISPR viruses were adjusted to 1 × 10^10^ infectious unit/mL just before use.

The AAV virus suspension (20 nl/side for mouse; 80 nl/side for rat) was bilaterally injected into either the caudal or rostral DMN using a NanoJect III® Programmable Nanoliter Injector (Drummond Scientific, Broomall, PA). The stereotaxic coordinates for the mouse caudal DMN were bregma − 7.60 mm, lateral 0.4 mm, depth 4.4 mm, and the mouse rostral DMN were bregma − 7.10 mm, lateral 0.4 mm, depth 4.35 mm. The coordinates for the rat caudal DMN were bregma − 14.0 mm, lateral 0.7 mm, depth 8.45 mm, and the rostral DMN were bregma − 13.2 mm, lateral 0.8 mm, depth 8.15 mm.

### Statistical analysis

All data were subjected to a Shapiro–Wilk normality test to ensure a normal distribution. Cell count and ELISA results were evaluated using either one-way ANOVAs or *t*-tests depending on the number of groups being compared. For data that did not have a normal distribution, a one-way ANOVA on ranks (or Mann–Whitney rank sum test for two-sample comparisons) was used. The Holm–Sidak method for multiple comparisons post hoc test was used to determine the data points with significant differences. Statistical analyses were performed using raw recorded data, prior to transformation into percent control for presentation. Data were considered significant at *p* < 0.05 and presented as mean ± standard error of the mean (SEM).

## Supplementary Information

**Additional file 1: Figure S1.** CARTp immunoreactivity does not colocalize with the sympathetic marker tyrosine hydroxylase. As sympathetic input into the spleen originates from the celiac ganglia, we examined if CARTp immunoreactivity is present in the celiac ganglia and could have traveled within splenic nerve fascicles to reach the spleen as has been reported for catecholaminergic fibers. A) Representative confocal images of CARTp immunoreactivity in the celiac ganglia. Although bisbenzamide staining revealed a large number of cells, no CARTp-positive cell bodies were detected. B) Drawing of the splenic artery showing the location of the splenic arterial branches. Splenic nerve fascicles travel within the adventitia of the splenic artery, entering the hilum of the spleen proximal to arterial branches. C) Representative image of tyrosine hydroxylaseimmunopositive nerve fascicles traveling within the adventitia of the splenic artery. D) To examine if these nerve fascicles carry CARTp-positive fibers into the spleen, we performed double-label immunohistochemistry for TH and CARTp. Representative images indicating that the nerve fascicles along the splenic artery are TH positive, but not CARTp immunopositive. No specific CARTp or TH immunoreactivities were observed when the primary antibodies were excluded from the staining procedure. **Figure S2.** The pAAV-TRE-tdTOMATO-WPRE and pAAV-Syn-tTA viruses do not cross synapses to induce tdTomato expression. A) Images of tdTomato and NeuN double-immunostained sections taken from the parabrachial nucleusand paraventricular nucleus, regions known to have monosynaptic connection to the DMN and NTS. tdTomato signal is seen in fibersbut not in the cell bodies of resident neurons. B) tdTomato immunoreactivitywas not detected in either the dorsal root gangliaor nodose ganglion, areas known to supply spleen innervation.

## Data Availability

All data generated or analyzed during this study are included in this published article (and its additional information files).

## References

[1] Moresco EM, LaVine D, Beutler B (2011). Toll-like receptors. Curr Biol.

[2] Savic S, Caseley EA, McDermott MF (2020). Moving towards a systems-based classification of innate immune-mediated diseases. Nat Rev Rheumatol.

[3] Pons V, Rivest S (2022). Targeting systemic innate immune cells as a therapeutic avenue for Alzheimer disease. Pharmacol Rev.

[4] Coutinho AE, Chapman KE (2011). The anti-inflammatory and immunosuppressive effects of glucocorticoids, recent developments and mechanistic insights. Mol Cell Endocrinol.

[5] Borovikova LV, Ivanova S, Zhang M, Yang H, Botchkina GI, Watkins LR, Wang H, Abumrad N, Eaton JW, Tracey KJ (2000). Vagus nerve stimulation attenuates the systemic inflammatory response to endotoxin. Nature.

[6] Whitaker AN (1969). Infection and the spleen: association between hyposplenism, pneumococcal sepsis and disseminated intravascular coagulation. Med J Aust.

[7] Ajmo CT, Vernon DO, Collier L, Hall AA, Garbuzova-Davis S, Willing A, Pennypacker KR (2008). The spleen contributes to stroke-induced neurodegeneration. J Neurosci Res.

[8] Rasouli J, Lekhraj R, Ozbalik M, Lalezari P, Casper D (2011). Brain-spleen inflammatory coupling: a literature review. Einstein J Biol Med.

[9] Martelli D, Yao ST, McKinley MJ, McAllen RM (2014). Reflex control of inflammation by sympathetic nerves, not the vagus. J Physiol.

[10] Bratton BO, Martelli D, McKinley MJ, Trevaks D, Anderson CR, McAllen RM (2012). Neural regulation of inflammation: no neural connection from the vagus to splenic sympathetic neurons. Exp Physiol.

[11] Bassi GS, Kanashiro A, Coimbra NC, Terrando N, Maixner W, Ulloa L (2020). Anatomical and clinical implications of vagal modulation of the spleen. Neurosci Biobehav Rev.

[12] Tracey KJ (2002). The inflammatory reflex. Nature.

[13] McAllen RM, McKinley MJ, Martelli D (2022). Reflex regulation of systemic inflammation by the autonomic nervous system. Auton Neurosci.

[14] Pereira MR, Leite PE (2016). The involvement of parasympathetic and sympathetic nerve in the inflammatory reflex. J Cell Physiol.

[15] Ma Q, Manaenko A, Khatibi NH, Chen W, Zhang JH, Tang J (2011). Vascular adhesion protein-1 inhibition provides antiinflammatory protection after an intracerebral hemorrhagic stroke in mice. J Cereb Blood Flow Metab.

[16] Sakaguchi R, Leiwe MN, Imai T: Bright multicolor labeling of neuronal circuits with fluorescent proteins and chemical tags. Elife. 2018;7:e40350. 10.7554/eLife.40350 PMC6245733 30454553

[17] Ferguson AV, Latchford KJ, Samson WK (2008). The paraventricular nucleus of the hypothalamus—a potential target for integrative treatment of autonomic dysfunction. Expert Opin Ther Targets.

[18] Chiang MC, Bowen A, Schier LA, Tupone D, Uddin O, Heinricher MM (2019). Parabrachial complex: a hub for pain and aversion. J Neurosci.

[19] Zingg B, Chou XL, Zhang ZG, Mesik L, Liang F, Tao HW, Zhang LI (2017). AAV-mediated anterograde transsynaptic tagging: mapping corticocollicular input-defined neural pathways for defense behaviors. Neuron.

[20] Shi XW, Jia F, Lyu P, Xu FQ (2022). A new anterograde trans-synaptic tracer based on Sindbis virus. Neural Regen Res.

[21] Zhang X, Renehan WE, Fogel R (2000). Vagal innervation of the rat duodenum. J Auton Nerv Syst.

[22] Skrzypczak-Wiercioch A, Salat K: Lipopolysaccharide-Induced Model of Neuroinflammation: Mechanisms of Action, Research Application and Future Directions for Its Use. Molecules 2022;27:5481. 10.3390/molecules27175481 PMC9457753 36080253

[23] Caravaca AS, Gallina AL, Tarnawski L, Tracey KJ, Pavlov VA, Levine YA, Olofsson PS (2019). An effective method for acute vagus nerve stimulation in experimental inflammation. Front Neurosci.

[24] Rosi S, Vazdarjanova A, Ramirez-Amaya V, Worley PF, Barnes CA, Wenk GL (2006). Memantine protects against LPS-induced neuroinflammation, restores behaviorally-induced gene expression and spatial learning in the rat. Neuroscience.

[25] Goehler LE, Gaykema RP, Hammack SE, Maier SF, Watkins LR (1998). Interleukin-1 induces c-Fos immunoreactivity in primary afferent neurons of the vagus nerve. Brain Res.

[26] Job MO, Kuhar MJ. Intraperitoneal administration of CART 55-102 inhibits psychostimulant-induced locomotion. J Drug Alcohol Res. 2012; 1. 10.4303/jdar/235601 PMC3659824 23705073

[27] Iliff JJ, Alkayed NJ, Golshani KJ, Weinstein J, Traystman RJ, West GA (2008). In vivo cerebrovascular effects of cocaine- and amphetamine-regulated transcript (CART) peptide. J Cardiovasc Pharmacol.

[28] Bellinger DL, Lorton D, Hamill RW, Felten SY, Felten DL (1993). Acetylcholinesterase staining and choline acetyltransferase activity in the young adult rat spleen: lack of evidence for cholinergic innervation. Brain Behav Immun.

[29] Buijs RM, van der Vliet J, Garidou ML, Huitinga I, Escobar C (2008). Spleen vagal denervation inhibits the production of antibodies to circulating antigens. PLoS ONE.

[30] Cailotto C, Costes LM, van der Vliet J, van Bree SH, van Heerikhuize JJ, Buijs RM, Boeckxstaens GE (2012). Neuroanatomical evidence demonstrating the existence of the vagal anti-inflammatory reflex in the intestine. Neurogastroenterol Motil.

[31] Guyot M, Simon T, Panzolini C, Ceppo F, Daoudlarian D, Murris E, Macia E, Abelanet S, Sridhar A, Vervoordeldonk MJ (2019). Apical splenic nerve electrical stimulation discloses an anti-inflammatory pathway relying on adrenergic and nicotinic receptors in myeloid cells. Brain Behav Immun.

[32] Chang L, Chen Y, Li J, Liu Z, Wang Z, Chen J, Cao W, Xu Y (2011). Cocaine-and amphetamine-regulated transcript modulates peripheral immunity and protects against brain injury in experimental stroke. Brain Behav Immun.

[33] Doran SJ, Ritzel RM, Glaser EP, Henry RJ, Faden AI, Loane DJ (2019). Sex differences in acute neuroinflammation after experimental traumatic brain injury are mediated by infiltrating myeloid cells. J Neurotrauma.

[34] Villapol S, Loane DJ, Burns MP (2017). Sexual dimorphism in the inflammatory response to traumatic brain injury. Glia.

[35] Campesi I, Montella A, Franconi F (2022). Human monocytes respond to lipopolysaccharide (LPS) stimulation in a sex-dependent manner. J Cell Physiol.

[36] Goverse G, Stakenborg M, Matteoli G (2016). The intestinal cholinergic anti-inflammatory pathway. J Physiol.

[37] Moreno A, Lopez LA, Fabra A, Arus C (1998). 1H MRS markers of tumour growth in intrasplenic tumours and liver metastasis induced by injection of HT-29 cells in nude mice spleen. NMR Biomed.

