# Supplementary material for: Caudal DMN neurons innervate the spleen and release CART peptide to regulate neuroimmune function
Source: J Neuroinflammation. 2023 Jul 4;20:158. doi: 10.1186/s12974-023-02838-2 (PMC10318820; doi:10.1186/s12974-023-02838-2)
Supplement: Supplementary file 1 — Additional file 1: Figure S1. CARTp immunoreactivity does not colocalize with the sympathetic marker tyrosine hydroxylase. As sympathetic input into the spleen originates from the celiac ganglia, we examined if CARTp immunoreactivity is present in the celiac ganglia and could have traveled within splenic nerve fascicles to reach the spleen as has been reported for catecholaminergic fibers. A) Representative confocal images of CARTp immunoreactivity in the celiac ganglia. Although bisbenzamide staining revealed a large number of cells, no CARTp-positive cell bodies were detected. B) Drawing of the splenic artery showing the location of the splenic arterial branches. Splenic nerve fascicles travel within the adventitia of the splenic artery, entering the hilum of the spleen proximal to arterial branches. C) Representative image of tyrosine hydroxylaseimmunopositive nerve fascicles traveling within the adventitia of the splenic artery. D) To examine if these nerve fascicles carry CARTp-positive fibers into the spleen, we performed double-label immunohistochemistry for TH and CARTp. Representative images indicating that the nerve fascicles along the splenic artery are TH positive, but not CARTp immunopositive. No specific CARTp or TH immunoreactivities were observed when the primary antibodies were excluded from the staining procedure. Figure S2. The pAAV-TRE-tdTOMATO-WPRE and pAAV-Syn-tTA viruses do not cross synapses to induce tdTomato expression. A) Images of tdTomato and NeuN double-immunostained sections taken from the parabrachial nucleusand paraventricular nucleus, regions known to have monosynaptic connection to the DMN and NTS. tdTomato signal is seen in fibersbut not in the cell bodies of resident neurons. B) tdTomato immunoreactivitywas not detected in either the dorsal root gangliaor nodose ganglion, areas known to supply spleen innervation. [file 12974_2023_2838_MOESM1_ESM.docx]

**Supplemental Information**

**
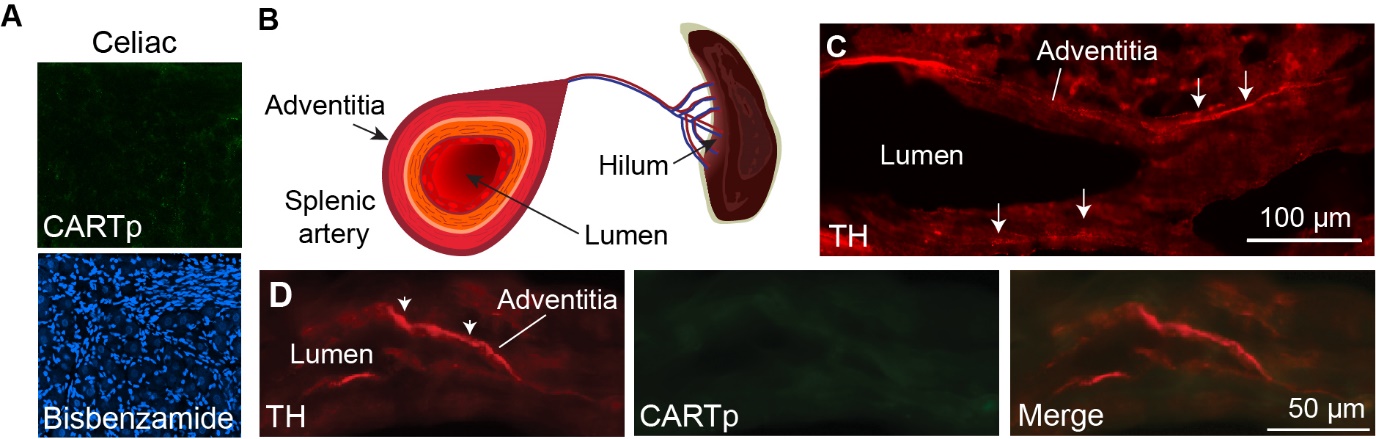
**

**Supplemental Figure 1. CARTp immunoreactivity does not colocalize with the sympathetic marker tyrosine hydroxylase (TH).** As sympathetic input into the spleen originates from the celiac ganglia, we examined if CARTp immunoreactivity is present in the celiac ganglia and could have traveled within splenic nerve fascicles to reach the spleen as has been reported for catecholaminergic fibers. **A)** Representative confocal images of CARTp immunoreactivity in the celiac ganglia. Although bisbenzamide staining revealed a large number of cells, no CARTp-positive cell bodies were detected. **B)** Drawing of the splenic artery showing the location of the splenic arterial branches. Splenic nerve fascicles travel within the adventitia of the splenic artery, entering the hilum of the spleen proximal to arterial branches. **C)** Representative image of tyrosine hydroxylase (TH, the rate-limiting enzyme for the synthesis of NE and a marker of sympathetic axons) immunopositive nerve fascicles traveling within the adventitia of the splenic artery. **D)** To examine if these nerve fascicles carry CARTp-positive fibers into the spleen, we performed double-label immunohistochemistry for TH and CARTp. Representative images indicating that the nerve fascicles along the splenic artery are TH positive, but not CARTp immunopositive. No specific CARTp or TH immunoreactivities were observed when the primary antibodies were excluded from the staining procedure (not shown).


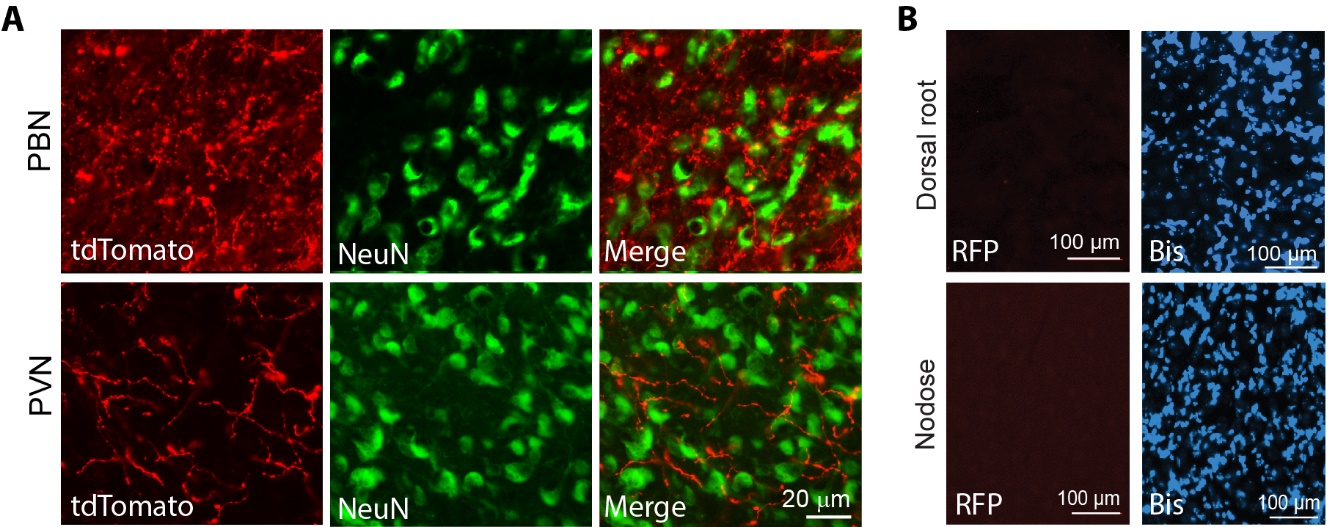


**Supplemental Figure 2. The pAAV-TRE-tdTOMATO-WPRE and pAAV-Syn-tTA viruses do not cross synapses to induce tdTomato expression. A)** Images of tdTomato and NeuN double-immunostained sections taken from the parabrachial nucleus (PBN) and paraventricular nucleus (PVN), regions known to have monosynaptic connection to the DMN and NTS. tdTomato signal is seen in fibers (presumed axons) but not in the cell bodies of resident neurons. **B)** tdTomato immunoreactivity (neither in fibers from the DMN, nor in resident cell bodies due to trans-synaptic infection) was not detected in either the dorsal root ganglia (T7-T10) or nodose ganglion, areas known to supply spleen innervation.
